# Periodontal inflamed surface area in patients on haemodialysis and peritoneal dialysis: a Croatian cross-sectional study

**DOI:** 10.1186/s12903-020-01086-7

**Published:** 2020-04-03

**Authors:** Bojana Križan Smojver, Karmela Altabas, Mladen Knotek, Nikolina Bašić Jukić, Andrej Aurer

**Affiliations:** 1 Department of Endodontics with Restorative Dentistry, Oral Medicine and Periodontology, Dental Clinic Zagreb, Perkovčeva ulica 3, 10000 Zagreb, Croatia; 2 Clinic of Internal Medicine, University Hospital Center “Sestre milosrdnice”, Vinogradska cesta 29, 10000 Zagreb, Croatia; 3 Clinic of Internal Medicine, University Hospital Center Merkur, Zajčeva ulica, 10000 Zagreb, Croatia; 4 Clinic of Internal Medicine, University Hospital Center Zagreb, Kišpatićeva ulica 12, 10000 Zagreb, Croatia; 5 Department of Periodontology, School of Dental Medicine, University of Zagreb, Gundulićeva ulica 5, 10000 Zagreb, Croatia

**Keywords:** Periodontitis, PISA, Peritoneal dialysis, Haemodialysis, Kidney failure

## Abstract

**Background:**

The decision to initiate dialysis treatment via haemodialysis (HD) or peritoneal dialysis (PD) often involves the consideration of complex factors and remains a matter of debate. The purpose of this study was to quantify the inflammatory burden that periodontitis causes in dialysis patients and to examine whether patients on PD and HD differ in terms of the periodontal inflamed surface area (PISA), which can be helpful for selecting the most appropriate dialysis modality.

**Methods:**

A cross-sectional study was performed on 58 consecutive patients on HD and 31 consecutive patients on PD. PISA was calculated using measurements of the clinical attachment level, recession and bleeding on probing. We performed the primary analysis using multivariable robust regression.

**Results:**

Patients on PD had a 746 mm^2^ (93%) lower mean PISA than patients on HD after adjustment for 20 possible confounders, including the duration of dialysis. The type of dialysis was independently correlated with the PISA (semipartial correlation: − 0.50; *p* = 0.017; false discovery rate < 5%). After adjusting for confounding factors, the correlation between the duration and type of dialysis was not significant (F (2,44) = 0.01; *p* = 0.994; η^2^ = 0.00). Differences in the PISA between patients who had undergone dialysis for less than a year, 2–3 years or ≥ 3 years were not significantly different in either of the two dialysis groups.

**Conclusions:**

PISA levels in Croatian patients on dialysis indicate a high need for periodontal treatment. PD is associated with a smaller PISA independent of many sociodemographic, lifestyle, laboratory and clinical factors. The duration of dialysis does not influence PISA levels.

**Trial registration:**

ISRCTN17887630.

A clinical study to investigate gum infection in patients undergoing kidney dialysis.

## Background

Chronic kidney disease (CKD) is defined as kidney damage or a glomerular filtration rate (GFR) of < 60 mL/min/1.73 m^2^ for 3 months or more, irrespective of the cause [1]. The loss of kidney functions leads to the accumulation of metabolic waste products that have an impact on the patient’s body. There are five CKD stages according to the estimated glomerular filtration rate (eGFR), and the last stage is end-stage renal disease (ESRD) or kidney failure (eGFR < 15 ml/min/1.73 m) [1]. Kidney transplantation is the treatment of choice for improved survival of ESRD patients, but when transplantation is not possible either due to the medical condition of the patient or lack of available organs, dialysis is a viable mode of treatment. There are two main types of dialysis: haemodialysis (HD) and peritoneal dialysis (PD). The decision to initiate dialysis treatment using haemodialysis or peritoneal dialysis is often complex and remains a matter of debate [2]. Although it has been shown that PD can provide similar or better survival rates and better quality of life and that PD is more economical than HD, in 2008, there were only 196,000 PD patients comprising 11% of the global dialysis population [3].

Periodontitis is a bacteria-driven chronic inflammatory disease that destroys the connective tissue and bone that support the teeth. Periodontitis represents a potential source of episodes of bacteraemia, especially in immune compromised patients [4]. Severe periodontitis is the 6th most prevalent disease worldwide, with an overall prevalence of 11.2% and approximately 743 million people affected [5].

Its impact on general health status is becoming increasingly apparent [6]. Periodontitis causes inflammation, systemic inflammatory responses or cross-reactivity that may lead to autoimmune reactions [7]. Researchers have reported that periodontitis poses an increased risk for various chronic diseases, such as coronary heart disease and stroke, diabetes, respiratory diseases and osteoporosis, as well as preterm low-birth-weight infants [8]. Furthermore, it contributes to systemic diseases due to an increased inflammatory burden [9]. Periodontitis can lead to systemic dispersal (via the bloodstream) of some locally produced pro-inflammatory mediators (e.g., IL-1b, IL-6 and TNF-α). Cytokines can stimulate the immune system, modify lipid metabolism and increase cytokine-mediated inflammatory processes (C-reactive protein), leading to further systemic conditions, such as endothelial dysfunction, atherosclerosis, coronary artery disease and glomerulonephritis. Moreover, it has been reported that periodontal bacteria can invade endothelial cells. A recent meta-analysis confirmed the association between CKD and periodontal disease and that the strength of this association was increased when severe periodontitis was considered [10]. Periodontitis thus represents an often overlooked problem in CKD patients. Lack of oral health management may contribute to systemic consequences, such as inflammation, infection, protein-energy wasting and atherosclerotic complications, which can contribute to increased morbidity and mortality [11]. The risk of systemic complications is probably higher if the surface of inflamed periodontal tissue is large. The periodontal inflamed surface area (PISA) represents the surface area in square millimetres of the bleeding pocket epithelium for all teeth. It is also advantageous for data processing and analysis because it can be treated as a continuous variable to quantify periodontal inflammation [12].

The aim of this study was to quantify the inflammatory burden that periodontitis poses in dialysis patients and to examine whether patients on PD and HD differ according to their PISA, which can be helpful in selecting the most appropriate dialysis modality. The main hypothesis put forth in this study is that PD is associated with a lower PISA.

## Methods

### Study design

A cross-sectional study including 58 consecutive patients on HD and 31 consecutive patients on PD was conducted. Patients were treated at the Department of Internal Medicine, University Hospital Center “Sestre milosrdnice”, Zagreb, Croatia, between February 2015 and January 2016. The study protocol was approved by the ethics committees of the Hospital and of the School of Dental Medicine, University of Zagreb. All patients gave written informed consent for participation. The study was performed in accordance with the World Medical Association Declaration of Helsinki [13].

### Participants

The targeted populations were patients of both genders, ≥18 years old, who were diagnosed with CKD (stage V) and treated with HD or PD. The exclusion criteria were an age < 18 years, level I-IV renal failure, renal transplant patients, having received periodontal treatment within the last 6 months, receiving antibiotic treatment at the time of examination and the presence of fewer than 8 teeth. We selected a consecutive sample of patients by the order of their dialysis initiation.

### Required sample size

The required sample size was calculated before the start of enrolment based on a pilot study performed on a sample of 20 patients on HD and 10 patients on PD during 2013. These patients were not included in the present study. A sample size of 70 patients achieved 80% power to detect an R^2^ ≥ 0.10 attributed to the type of dialysis using an F-test with a significance level of *p* ≤ 0.05, after adjusting for 15 possible confounders whose association with PISA was R^2^ = 0.20. The ratio of the size of the two groups was determined to be 2:1 with respect to the sizes of the populations of patients treated with the two types of dialysis. Expecting up to 20% of responders to have missing data, the initially needed sample size was determined to be *n* = 74. A power analysis was performed using PASS 14 Power Analysis and Sample Size Software (2015; NCSS, LLC, Kaysville, Utah, USA).

### Outcomes

The main outcome was PISA, which was calculated based on BOP, CAL and REC measurements that were performed at six sites on each tooth using a periodontal probe (PCP 15; Hu-Friedy, Chicago, IL, USA). All periodontal examinations were performed by the same calibrated examiner.

PISA was calculated by the on-line calculator available at www.parsprototo.info [12]. Using the formulas described by Hujoel et al. [14], a Microsoft Excel spreadsheet was constructed to facilitate the PISA calculation [12]. PISA was calculated in seven steps: 1) after inputting into Excel the CAL measurements at six sites per tooth, the mean CAL for each particular tooth was calculated; 2) the mean CAL around a particular tooth was entered into the appropriate formula for the translation of linear CAL measurements to the attachment loss surface area (ALSA) for that specific tooth; 3) after filling in the REC measurements at six sites per tooth, the mean REC was calculated for each particular tooth; 4) the mean REC around a particular tooth was entered into the appropriate formula for the translation of linear REC measurements to the REC surface area (RSA) for that tooth; 5) the RSA for the specific tooth was subtracted from the ALSA of that tooth, rendering the periodontal epithelial surface area (PESA) of the tooth (PESA = ALSA-RSA); 6) the PESA for the tooth was then multiplied by the proportion of sites around the tooth affected by BOP; for example, if three of the maximum of six sites were affected by BOP, the PESA of that particular tooth was multiplied by 3/6, thereby rendering the PISA for the tooth; and 7) the sum of all individual PISAs around individual teeth was calculated, amounting to the total PISA within a patient’s mouth [7]. The independent variable was HD or PD.

### Possible confounding variables controlled by the multivariable analysis

Based on the literature and previous studies, before the start of analysis, 15 variables were selected with possible confounding effects: 1) age; 2) duration of dialysis in months; 3) number of teeth; 4) smoking; 5) C-reactive protein; 6) dialysis adequacy measured by the ratio between dialyser clearance (K) (mL/min) multiplied by time in minutes (t) and the volume of water a patient’s body contains (Kt/V); 7) thrombocytes; 8) urea; 9) phosphorus; 10) high-density lipoprotein (HDL) cholesterol; and treatment with 11) beta-blockers, 12) angiotensin-converting enzyme inhibitors, 13) central α-2 receptor agents, 14) angiotensin II AT1-receptor blockers, and 15) α_1_-adrenoceptor antagonists. We also controlled five additional possible confounders: diabetes mellitus, duration of diabetes, glycated haemoglobin (HbA1c), leukocytes, and the last visit to the dentist.

### Other variables used to describe the two samples

Other variables used to describe the two samples were gender; education; alcohol consumption; body mass index (kg/m^2^); arterial hypertension; dry mouth; number of daily tooth-brushing episodes; usage of interdental brushes/floss; self-reported bleeding gums; treatment with calcium channel blocker, antiplatelet or immunosuppressive medication; parathyroid hormone; potassium; mean corpuscular volume; total cholesterol; LDL cholesterol, triglycerides; erythrocytes; creatinine; serum albumin; sodium; alkaline phosphatase; haematocrit; and haemoglobin. Information about age, gender, education, smoking habits and consumption of alcohol, xerostomia, self-observed bleeding of gums and oral hygiene habits was obtained by a questionnaire (Additional file 1) that was designed specifically for this study. The data on other variables were obtained from hospital medical records. We did not independently assess the validity and reliability of the medical records data.

### Statistical analysis

The introductory, bivariable analysis was performed using analysis of variance, with Hedge’s g and 95% confidence intervals presented as the measure of the standardized effect size, and the Mann-Whitney U test. The hypothesis was tested using robust regression and iteratively reweighted least squares simultaneously on all 20 variables and the type of dialysis. We checked the main multivariable analysis results using quantile regression and the adjusted medians of PISA. A main multivariable analysis was performed on the per protocol population with list-wise deletion of patients with missing data. Unstandardized and standardized regression coefficients were presented with the t-test statistic with n-p-1 degrees of freedom, where p is the total number of parameters in the model, two-tailed statistical significance of multivariable regression coefficients, zero-order Pearson product-moment correlation with PISA (r), and semipartial correlation with PISA after controlling for all other variables (sr). Imputation of missing data was performed only for the sensitivity analysis. Multiple imputation was applied with fully conditional specification of the iterative Markov chain Monte Carlo method (data augmentation algorithm) with a maximum of 100 iterations. To minimize the random fluctuation effect, 30 imputed data sets were created. As the HbA1c value was not available for patients who were not diagnosed with diabetes mellitus, we imputed the value of 5.0. We performed a sensitivity analysis by imputing the values of HbA1c = 4.0 and 7.0. The inflation of false positives was controlled using the Benjamini-Holchberg method with the false discovery rate (FDR) set at ≤5%. The level of statistical significance was set to two-tailed *p* < 0.05, and all confidence intervals were set to 95%. The statistical analysis was performed using the R Development Core Team [15].

## Results

**Fig. 1:**
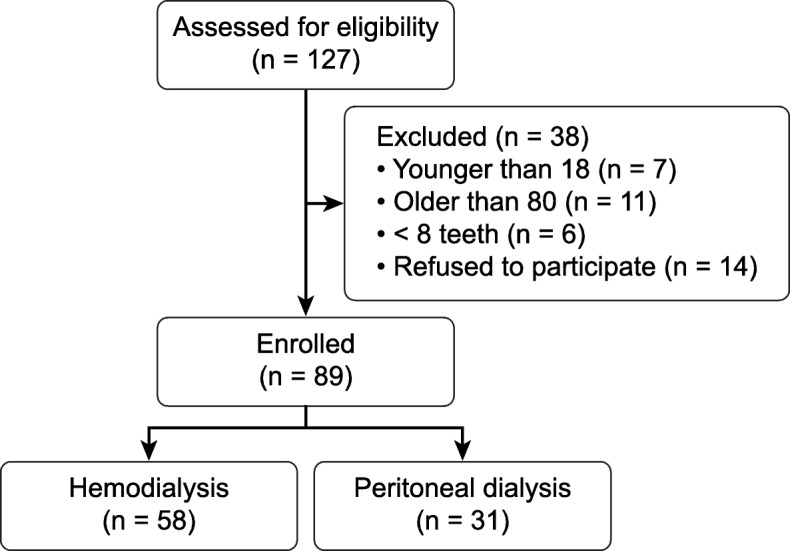
Patient flow diagram

After screening 127 patients, 89 were enrolled, 58 of whom were on HD and 31 of whom were on PD (Fig. 1). The two groups were different with regard to many sociodemographic, vital, lifestyle and clinical characteristics (Table 1). Patients on HD were older, had been on dialysis longer, had fewer teeth, and had less self-reported bleeding of the gums. According to blood count, the largest differences were in Kt/V and parathyroid hormone levels. Kt/V was higher in the PD group, and parathyroid hormone was higher in the HD group. There were also relevant differences in C-reactive protein values (higher in the HD group) and in thrombocyte (higher in the PD group) (Table 2).

**Table 1:** Patients’ sociodemographic, vital, lifestyle and clinical characteristics

	Dialysis			
	HD (*n* = 58)		PD (*n* = 31)	
Age (years), median (IQR)	65	(59–76)	49	(39–60)
Gender				
Men	31	(53.4)	16	(51.6)
Women	27	(46.6)	15	(48.4)
Education				
Primary	8	(14.5)	3	(11.5)
Secondary	35	(63.6)	19	(73.1)
University	12	(21.8)	4	(15.4)
Smoking of tobacco				
Non-smoker	24	(42.9)	16	(61.5)
Ex-smoker	27	(48.2)	6	(23.1)
Current smoker	5	(8.9)	4	(15.4)
Alcohol consumption	8	(14.8)	8	(29.6)
Body mass index (kg/m^2^), median (IQR)	25	(22–29)	26	(22–28)
Diabetes mellitus	24	(41.4)	8	(26.7)
Duration of diabetes mellitus, median (IQR)	11	(7–16)	12	(9–23)
HbA1c, median (IQR)	6.2	(5.9–6.7)	6.1	(5.8–6.9)
Arterial hypertension	48	(82.8)	30	(100.0)
Duration of dialysis (months); median (IQR)	36	(13–73)	12	(8–27)
Dry mouth				
No	24	(42.9)	13	(48.1)
Periodically	23	(41.1)	10	(37.0)
Permanently	9	(16.1)	4	(14.8)
Number of tooth-brushing episodes daily				
Do not brush	3	(5.4)	0	(0.0)
1	14	(25.0)	10	(37.0)
2	30	(53.6)	13	(48.1)
≥ 3	9	(16.1)	4	(14.8)
Usage of an interdental brush/floss	15	(26.8)	8	(29.6)
The last visit to the dentist				
within the last year	31	(60.8)	18	(72.0)
2–3 years ago	13	(25.5)	5	(20.0)
≥ 4 years ago	7	(13.7)	2	(8.0)
Bleeding gums	5	(8.9)	7	(26.9)
Number of teeth, median (IQR)	16	(9–20)	23	(17–28
Antihypertensive therapy				
Angiotensin-converting enzyme inhibitors	17	(30.4)	3	(13.0)
Beta-blockers	38	(67.9)	12	(52.2)
Calcium channel blockers	27	(48.2)	11	(47.8)
Angiotensin II AT1-receptor blockers	10	(17.9)	1	(4.8)
Central α-2 receptor agents	10	(17.9)	11	(47.8)
α_1_-adrenoceptor antagonists	22	(39.3)	3	(13.0)
Antiplatelet therapy	13	(23.2)	5	(21.7)
Immunosuppressive therapy	2	(3.6)	1	(7.7)

Data are presented as the number (percentage) of participants if not stated otherwise

Data were missing for education in 8 (9.0%), smoking in 7 (7.9%), alcohol in 8 (9.0%), diabetes mellitus in 1 (1.1%), arterial hypertension in 1 (1.1%), body mass index in 12 (13.5%), duration of dialysis in 2 (2.2%), dry mouth in 6 (6.7%), number of tooth-brushing episodes daily in 6 (6.7%), usage of an interdental brush/floss in 6 (6.7%), the last visit to the dentist in 13 (14.6%), and bleeding gums in 7 (7.9%) participants

*Abbreviations*: *IQR* Interquartile range, *HbA1c* Glycated haemoglobin

**Table 2:** Laboratory parameters; only patients with complete data were included (*n* = 65; patients with missing data, *n* = 24 (27%)

	Dialysis			
	HD (*n* = 58)		PD (*n* = 31)	
Kt/V	1.4	(1.3–1.5)	1.9	(1.8–2.1)
Parathyroid hormone (pmol/L)	243	(100–428)	32	(19–80)
C-reactive protein (mg/L)	4.8	(2.0–14.1)	1.6	(0.8–3.4)
Thrombocytes (10^9^/L)	180	(141–209)	240	(183–326)
Potassium (mmol/L)	4.7	(4.1–5.1)	4.5	(4.1–4.7)
Mean corpuscular volume (fL)	94	(92–96)	91	(88–93)
Leukocytes (10^9^/L)	6.0	(5.1–7.8)	7.3	(6.2–8.9)
Total cholesterol (mmol/L)	4.1	(3.6–4.9)	4.9	(3.9–6.1)
HDL cholesterol (mmol/L)	1.1	(0.9–1.4)	1.5	(1.0–1.9)
Erythrocytes (10^12^/L)	3.5	(3.1–3.7)	3.7	(3.3–4.1)
Creatinine (μmol/L)	712	(614–814)	786	(655–893)
Phosphorus (mmol/L)	1.5	(1.3–1.8)	1.6	(1.4–1.9)
Triglycerides (mmol/L)	1.4	(0.9–2.1)	1.7	(1.1–2.7)
Urea (mmol/L)	19.8	(15.8–22.4)	21.0	(17.4–25.4)
Serum albumin (g/L)	38	(36–40)	39	(37–40)
Sodium (mmol/L)	136	(134–138)	138	(134–139)
Alkaline phosphatase (g)	79	(64–108)	89	(69–112)
Haematocrit (L/L)	0.33	(0.30–0.35)	0.34	(0.31–0.36)
LDL cholesterol (mmol/L)	2.3	(1.7–2.9)	2.6	(1.5–3.6)
Haemoglobin (g/L)	111	(101–117)	109	(101–117)

Data are presented as the median (interquartile range) if not stated otherwise

Data were missing for erythrocytes in 2 (2.2%), haemoglobin in 2 (2.2%), haematocrit in 20 (22.5%), mean corpuscular volume in 2 (2.2%), leukocytes in 13 (14.6%), thrombocytes in 2 (2.2%), urea in 3 (3.4%), creatinine in 3 (3.4%), Kt/V in 15 (16.9%), C-reactive protein in 4 (4.5%), albumin in 4 (4.5%), total cholesterol in 10 (11.2%), HDL cholesterol in 10 (11.2%), LDL cholesterol in 10 (11.2%), triglycerides in 10 (11.2%), potassium in 2 (2.2%), sodium in 19 (21.3%), phosphorus in 3 (3.4%), alkaline phosphatase in 19 (21.3%), and parathyroid hormone in 8 (9.0%) participants

*Abbreviations*: *Kt/V* Dialysis adequacy measured as the ratio between dialyser clearance (K) (mL/min) multiplied by time in minutes (t) and volume of water a patient’s body contains; *HDL* High density lipoprotein cholesterol, *LDL* Low density lipoprotein cholesterol

**Fig. 2:**
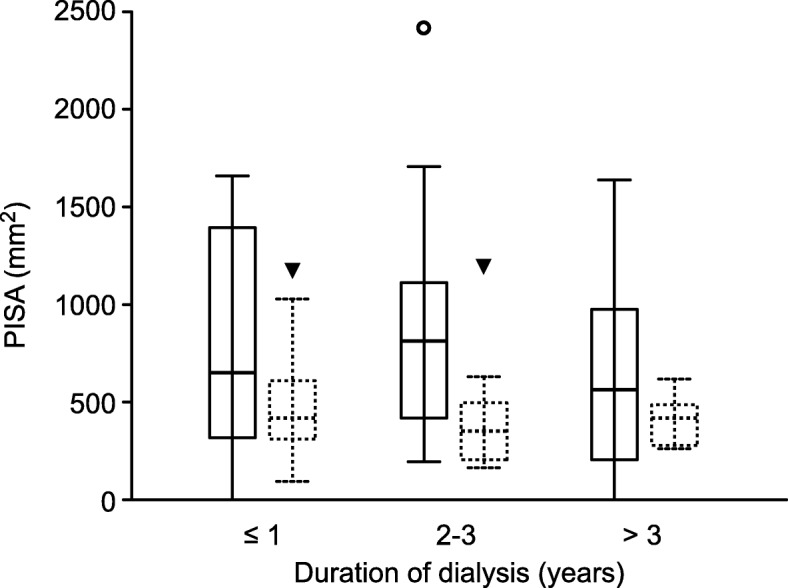
PISA by the type and duration of dialysis. The solid line represents HD; the dotted line represents PD; the middle line represents the median; the boxes represent the interquartile range; the circle outside the maximum lines represents patients on HD with PISA values larger than the upper quartile plus 1.5 times the interquartile range; the black triangle represents the same for patients on PD; only patients with complete data were included (*n* = 65; patients with missing data, *n* = 24 (27%)

In the introductory bivariable analysis, PISA was significantly different between the two dialysis groups. The mean PISA (SD) was 738 (520.4) mm^2^ in patients on HD and 470 (277.8) mm^2^ in patients on PD (F(1,87) = 7.12; *p* = 0.009; Hedge’s g = 0.59; 95% CI − 1.04 to − 0.15; FDR < 5%). The median PISA (IQR) was 624 (335–1042) mm^2^ in patients on HD and 401 (272–605) mm^2^ in patients on PD (Mann-Whitney test, U = 634; z = − 2.28; *p* = 0.022; the probability that patients on HD had larger PISA values than patients on PD was 65%). After adjusting for 20 confounding factors, the type of dialysis was found to be significantly associated (FDR < 5%) with PISA (Table 3). Patients on PD had a significantly lower PISA. After adjusting for 20 confounding factors, the mean (95% CI) PISA was 798 (681–914) mm^2^ in the HD group and 52 (0–417) mm^2^ in the PD group. This 746 mm^2^ absolute difference represented a 93% relative difference. The adjusted median PISA was 732 mm^2^ in the HD group and 190 mm^2^ in the PD group. This 542 mm^2^ absolute difference represented a 74% relative difference. The type of dialysis showed a semipartial correlation with PISA (sr = − 0.50, *p* < 0.017; FDR < 5%). The variation in HbA1c values imputed for patients with no diagnosed diabetes mellitus 4, 5 and 7 revealed identical results. A sensitivity analysis was performed by multiple imputation of the missing data of 20 confounding factors. A pooled analysis on 30 data sets with complete (imputed) data revealed very similar results to the result of the per-protocol and complete case (listwise deletion) analyses: patients on PD had a mean (95% CI) PISA of − 613 (− 995 to − 232); robust regression, *p* = 0.002) and a median (95% CI) PISA of − 448 (− 887 to − 9; quantile regression, *p* = 0.046). PISA was significantly lower in the PD group regardless of the duration of dialysis (Fig. 2). After adjusting for confounding factors, the interaction between the duration and type of dialysis was not significant (F (2,44) = 0.01; *p* = 0.994; η^2^ = 0.00). Differences in PISA between patients who had been dialysed for less than a year, 2–3 years or ≥ 3 years were not significantly different in any of the two dialysis groups.

**Table 3:** Robust regression on PISA; variables were simultaneously input; only patients with complete data were included

	b	Β	t	p	FDR < 5%	r	sr
Dialysis							
HD (referent group)							
PD	−526.8	−0.53	−2.50	0.017	✓	−0.31	− 0.50
Confounders that were controlled							
Age (years)	−2.0	−0.05	−0.47	0.644		−0.00	−0.02
Duration of dialysis (months)	−0.3	−0.03	− 0.18	0.856		0.08	−0.12
Number of teeth	43.3	0.77	5.95	< 0.001	✓	0.52	0.74
The last visit to the dentist	−13.4	−0.11	−0.99	0.327		−0.06	0.03
Smoking							
Non-smoker (referent group)							
Ex-smoker	47.3	0.10	0.33	0.744		−0.12	−0.11
Current smoker	163.7		0.86	0.393			
Diabetes mellitus	− 418.0	−0.43	−1.73	0.092		−0.09	−0.26
Duration of diabetes mellitus	27.2	0.41	1.91	0.064		−0.08	0.32
HbA1c^a^	−44.1	−0.10	−0.69	0.495		−0.14	−0.04
C-reactive protein (mg/L)	−9.5	−0.28	−2.12	0.041		−0.15	−0.42
Kt/V	− 494.5	−0.36	−2.20	0.034	✓	−0.21	−0.28
Thrombocytes (10^9^/L)	−2.2	−0.35	−1.80	0.081		−0.11	−0.04
Leukocytes (10^9^/L)	22.5	0.12	0.78	0.442		−0.06	− 0.06
Urea (mmol/L)	−4.9	−0.06	− 0.43	0.671		0.03	−0.15
Phosphorus (mmol/L)	138.4	0.11	0.94	0.354		0.07	0.22
HDL cholesterol (mmol/L)	−61.7	−0.08	−0.38	0.707		−0.07	0.01
Antihypertensive therapy							
Beta-blockers	−209.4	−0.22	−1.64	0.110		0.22	−0.19
Angiotensin-converting Enzyme inhibitors	− 216.5	−0.20	− 1.66	0.106		0.07	0.03
Central α-2 receptor agents	11.0	0.02	0.07	0.943		0.16	0.10
Angiotensin II AT1-receptor blockers	− 159.1	−0.11	− 1.16	0.256		0.01	0.03
α_1_-adrenoceptor antagonists	− 188.5	−0.18	−1.47	0.151		0.10	−0.31

*Abbreviation*: *b* Unstandardized multivariable robust regression coefficient, *β* Standardized multivariable robust regression coefficient, *t* Student’s t-test statistic with n-p-1 degrees of freedom where p is total number of parameters in the model, *p* two-tailed test, statistical significance of multivariable regression coefficient, *FDR* False discovery rate of < 5% calculated using the Benjamini-Hochberg method, *r* zero-order Pearson product-moment correlation with PISA, *sr* semipartial correlation with PISA after controlling for all other variables, *HbA1c* Glycated haemoglobin, *Kt/V* Dialysis adequacy measured as the ratio between dialyser clearance (K) (mL/min) multiplied by time in minutes (t) and volume of water a patient’s body contains, *HDL* High-density lipoprotein cholesterol

^a^HbA1c = For participants not diagnosed with diabetes, the HbA1c value was arbitrarily set to 5.0

## Discussion

This study confirmed the hypothesis that PD is associated with a smaller PISA than HD, indicating a smaller inflammatory burden, independent of a large number of sociodemographic, lifestyle, laboratory and clinical factors. This result may be due to the fact that HD is better for the removal of small-molecular-weight molecules, such as urea and creatinine, which are not real uremic toxins (Kt/V is higher in patients on HD than in patients on PD), and that PD is better for the removal of mid-sized molecules (uremic toxins). Uremic toxins can lower the capacity of the immune system [16–18], which may result in a better immune response and healthier periodontal tissue in PD patients. As all dialysis patients are potential organ recipients, it is of upmost importance that there is no hidden source of inflammation prior to kidney transplantation. A meta-analysis published in 2016 [19] also reported that pretransplant dialysis influences short- and long-term complications after kidney transplantation and that PD may be a better choice of pretransplant dialysis modality than HD.

This study also showed the degree of inflammatory burden in Croatian dialysis patients presented by PISA. After adjusting for all confounders, the mean PISA was 798 mm^2^ in the HD group and 52 mm^2^ in the PD group. According to the PISA cut-off values defined by the Centers for Disease Control and Prevention-American Academy of Periodontology for the classification of periodontitis, i.e., that severe periodontitis ranges from 934,71 mm^2^ to 3274,96 mm^2^, moderate periodontitis ranges from 521,58 to 790,30 mm^2^, mild periodontitis ranges from 110,16 and 447,01 mm^2^, and no periodontitis corresponds to PISA values from 10.22 mm^2^ to 62.78 mm^2^ [20], periodontal treatment is highly needed among dialysis patients in Croatia.

To our knowledge, this is the first study to measure or compare PISA in CKD patients on HD and PD. Some previous studies have compared other periodontal indices among patients on HD and PD. Cross-sectional studies conducted in Brazil, Canada, Turkey, USA and Taiwan have reported that chronic severe periodontitis is significantly more prevalent among patients on HD than among healthy persons, and periodontal disease is comparatively more prevalent and more severe in CKD patients [21–25]. However, PISA provides important advantages over these other periodontal indices. It represents a classification that quantifies the amount of inflamed periodontal tissue and, as such, quantifies the systemic inflammatory burden [7, 12].

Previous studies [26] have shown higher levels of periodontitis in patients with CKD than in healthy controls, and the disease is most advanced in maintenance HD patients but successively diminished in PD and pre-dialysis CKD patients.

In 2016, Chinese authors [27] stated that the periodontal status of HD and PD patients was worse than that in healthy controls, but there were no statistically significant differences between the PD and HD groups. However, the average calculus surface index was significantly higher in HD patients than in PD patients. This finding may be related to the alteration in serum phosphorus-calcium in HD patients.

Bayraktar et al. [28] reported a higher gingival index (GI) in the HD group than in the peritoneal group and higher calculus accumulation in both dialysis groups compared with healthy controls. Brito et al. [29] concluded that patients on PD had similar CALs to healthy controls. Moreover, according to Thorman et al. [30], pre-dialysed patients and patients on HD have a higher prevalence of severe periodontitis than healthy controls and patients on PD. Such results can also be explained by the psychological state of patients according to dialysis type and satisfaction with their quality of life. Patients on HD visit the hospital several times a week and are connected to dialysis machines for approximately 4 h. Patients on PD can perform dialysis procedures in their own home or at other clean locations, have more independence and are able to more actively work; and consequently, patients on PD report a better quality of life. In fact, better quality of life and higher satisfaction of patients on PD have been statistically proven by various studies [31, 32]. Some studies have reported high levels of quality of life among patients on PD, although these levels were not statistically significant from the quality of life of other dialysis patients [33–35]. It has been proposed that patients on PD exhibit higher motivation and have a more proactive approach regarding their oral hygiene habits, thus leading to a better periodontal state.

This study did not find significant differences in PISA according to the duration of dialysis in the three groups of patients: those on dialysis for less than a year, for 2–3 years and for more than 3 years. These results are in accordance with the study reported by Parkar and Ajithkrishnan [36], which outlined four subgroups according to the duration of dialysis: less than 3 months, 4–6 months, 7–9 months and 10–12 months. The authors of that study reported no effect of the duration of dialysis on periodontal tissues. A similar study by Marakoglu et al. [37] also revealed no significant differences in age, gingival index, plaque index or periodontal pocket depth among subgroups of patients on HD for less than 1 year, 1–3 years and more than 3 years. In contrast, Cengiz et al. [38] compared patients on dialysis for less than 5 years, 5–10 years and more than 10 years and concluded that there were significant increases in plaque index, gingival bleeding and periodontal pocket depth after 5 years and that the difference was statistically more significant after 10 years. These findings suggest that the significant influence of dialysis on periodontal health becomes obvious after 5 years.

### Limitations of this study

The first limitation of this study is that the patients were not randomly allocated to receive HD or PD. An attempt was made to control this source of bias by controlling the effect of a larger number of possible confounders. Nevertheless, we were able to control only the included and known variables, while different important unmeasured factors remained uncontrolled. Periodontal status before the initiation of dialysis and the duration of kidney disease are likely to be highly important factors. The cross-sectional design of this study prevented the observation of a temporal sequence between periodontitis and dialysis, and for this reason, it was not possible to make any causal inferences. Second, patients were enrolled in the specialized nephrology ward of a large university teaching hospital in a highly urban area of the country’s capital. It is possible that periodontal disease, dialysis parameters, and their association are different in small, regional hospitals with sparser resources and less educated patients of a lower socioeconomic status. There is no evidence on which to base this claim, but the possibility should be taken into account. Therefore, these findings should not be uncritically generalized to the general Croatian population of patients treated for CKD. Third, the primary outcome was not independently assessed, and the participating investigators were not blinded to the type of dialysis. These factors likely induced bias against the null hypothesis. Therefore, these findings are probably somewhat overoptimistic and should be replicated in properly blinded studies. Fourth, the dosages of monitored therapies were not controlled, whereas only whether or not the patient was treated with a particular drug was monitored. Fifth, a consecutive sample of patients was selected, which might have increased the risk of selection bias.

## Conclusions

Patients on PD and HD in Croatia have poor periodontal conditions, presenting high PISA values, and require periodontal treatment. PD is associated with a smaller PISA independent of many sociodemographic, lifestyle, laboratory and clinical factors. No relationship between the duration of dialysis and PISA was found. A prospective, randomized, control study is needed to test for a causal relationship.

## Supplementary information

**Additional file 1.** Oral hygiene habits, alcohol consumption and smoking habits questionnaire

## Data Availability

All data generated from this study are available from the corresponding author upon reasonable request.

## Abbreviations

PD: Peritoneal dialysis
HD: Haemodialysis
PISA: Periodontal inflamed surface area
CKD: Chronic kidney disease
eGFR: Estimated glomerular filtration rate
ESRD: End-stage renal disease
CAL: Clinical attachment level
REC: Recession
BOP: Bleeding on probing
ALSA: Attachment loss surface area
REC: Recession surface area
PESA: Periodontal epithelial surface area
HDL: High-density lipoprotein
LDL: Low-density lipoprotein
FDR: False discovery rate
IQR: Interquartile range
Kt/V: Fractional urea clearance
HbA1c: Glycated haemoglobin
