# Supplementary material for: Periodontal inflamed surface area in patients on haemodialysis and peritoneal dialysis: a Croatian cross-sectional study
Source: BMC Oral Health. 2020 Apr 3;20:95. doi: 10.1186/s12903-020-01086-7 (PMC7118952; doi:10.1186/s12903-020-01086-7)
Supplement: Supplementary file 1 — Additional file 1. Oral hygiene habits, alcohol consumption and smoking habits questionnaire [file 12903_2020_1086_MOESM1_ESM.docx]

**Oral hygiene habits, alcohol consumption and smoking habits questionnaire**

Name: ___________________________

Age: ___________________________

Gender: 🞎 Male

🞎 Female

Education: 🞎 Primary or below

🞎 Secondary

🞎 College or above

Do you smoke cigarettes? 🞎 Yes

🞎 I am a former smoker

🞎 No

If you do smoke, how many cigarettes per day? _____________

Do you drink alcohol? 🞎 No

🞎 Occasionally

🞎 Yes, up to three times a week

🞎 Yes, three or more times a week

Do you feel dryness of the mouth? 🞎 Yes

🞎 Occasionally

🞎 No

When was the last time you visited the dentist? ____________

How often do you brush your teeth? 🞎 Never / irregularly

🞎 Once a day

🞎 Twice a day

🞎 Three times a day or more

Do you use interdental oral hygiene aids such as interdental brushes or floss? 🞎 Yes

🞎 No

Do your gums bleed while brushing? 🞎 Yes

🞎 Occasionally

🞎 No
